# *Longivena*, a new robber-fly genus from Brazil (Diptera, Asilidae, Asilinae)

**DOI:** 10.3897/zookeys.443.8324

**Published:** 2014-09-30

**Authors:** Rodrigo Vieira, José Albertino Rafael

**Affiliations:** 1 Instituto Nacional de Pesquisas da Amazônia – INPA, Coordenação de Biodiversidade, CEP 69060–000, Manaus, Amazonas, Brazil; 2 Instituto Nacional de Pesquisas da Amazônia – INPA, Coordenação de Pesquisas em Biodiversidade, Campus II, CEP 69060–000, Manaus, Amazonas, Brazil

**Keywords:** Asilidae, Neotropical, taxonomy, new genus and species, *Efferia* group

## Abstract

*Longivena* **gen. n.** and five new species are described and illustrated from caatinga and cerrado habitats from Brazil: *Longivena digitata* **sp. n.**, type–species (Maranhão, Bahia, Minas Gerais and Mato Grosso do Sul states), *Longivena bilobata* **sp. n.** (Maranhão state), *Longivena flava* **sp. n.** (Mato Grosso do Sul state), *Longivena limeiraoliverai* **sp. n.** (Maranhão state), *Longivena spatulata* **sp. n.** (Maranhão state). An illustrated key is also provided.

## Introduction

There are 178 currently known genera of Asilinae Latreille, 1802, of which 67 are known from the Neotropical region and 19 from Brazil (Vieira 2012a, Artigas and Vieira 2014, Vieira and Ayala 2014). Due to the great similarity found between members of Asilinae, some artificial generic groups have been proposed to fascilitate identification (Artigas and Papavero 1997a). These groups were established based mainly on male and female terminalia, since external characters are insufficient to separate genera.

For the Nearctic and Neotropical regions, 11 groups have been proposed (Artigas and Papavero 1995a, b, c, d, e, f, g; 1997a, b, c): *Asilus* (Asilini *sensu stricto*) group, *Efferia* group, *Eicherax* group, *Eichoichemus* group, *Glaphyropyga* group, *Lecania* group, *Lochmorhynchus* group, *Mallophora* group, *Myaptex* group, *Ommatius* group and *Proctacanthus* group.

In this paper, a new robber fly genus, *Longivena* gen. n., is proposed. This genus is morphologically similar to the genera of the artificial group *Efferia* and is characterized by the r3 cell closed and petiolate and the long stump vein (supernumerary crossvein) on R_4_ reaching the base of R_2+3_. This paper presents a description, diagnoses, illustrations of a new genus of Asilinae, and comments on related genera.

## Material and methods

This study is based on the examination of specimens housed in the following institutions: CZMA – Coleção Zoológica do Maranhão, Universidade Estadual do Maranhão, Caxias, Maranhão state, Brazil (Dr Francisco Limeira-de-Oliveira) and Instituto Nacional de Pesquisas da Amazônia (INPA), Manaus, Amazonas state, Brazil (Dr Marcio Oliveira). Morphological terminology follows Cumming and Wood (2009). Vieira’s (2012b) techniques were used to examine the terminalia. After examination and illustration, the detached parts were placed in microvials with glycerin and pinned along the same pin with their respective specimen. Standard measurements were taken utilizing a filar micrometer. Label data is cited in full, with the original spellings, punctuations, and dates. Information presented within square brackets is complementary data not included on the labels. Data for the same specimen but from different labels are separated by slashes (/). The map was generated with software SimpleMappr.

## Taxonomy

### Longivena gen. n.

Taxon classificationAnimaliaDipteraAsilidae

http://zoobank.org/49AE8CEA-8129-485B-AAE7-506F03ECC168

#### Type species.

*Longivena digitata* sp. n. (present designation).

#### Etymology.

From Latin, *longi* = long + *vena*= vein, referring to the long stump vein (supernumerary crossvein) on R_4_ that reaches the base of R_2+3_.

#### Gender.

Feminine.

#### Distribution.

**Brazil:** Maranhão, Bahia, Minas Gerais and Mato Grosso do Sul states (Fig. 50).

#### Description.

**Figures 1–9.**
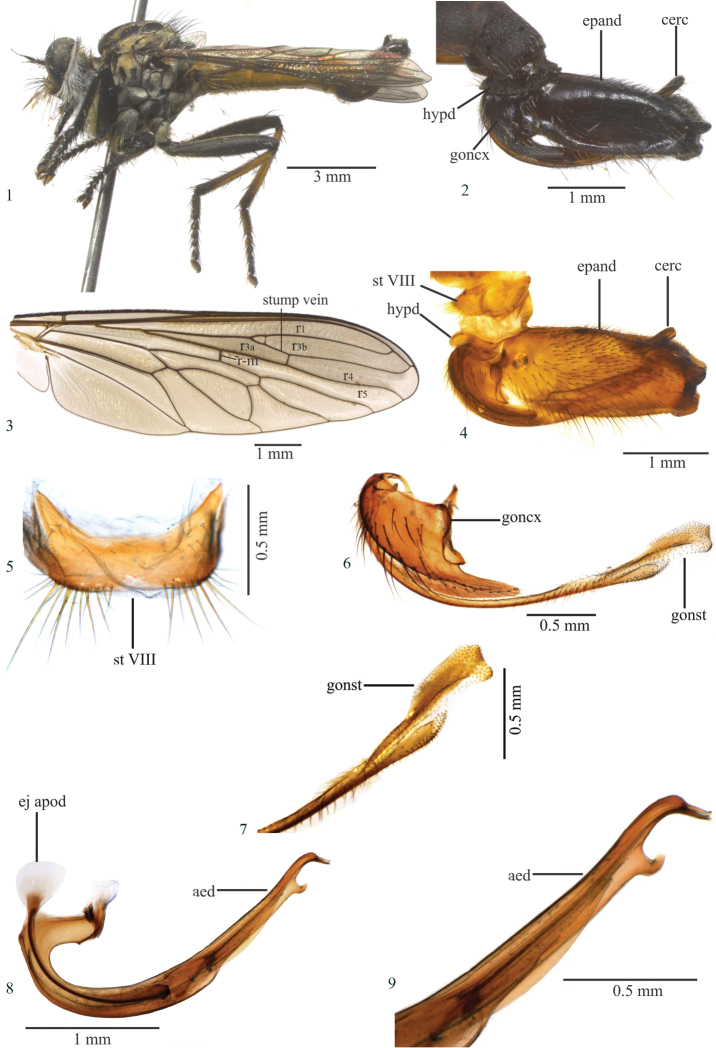
*Longivena bilobata* sp. n. Holotype male. **1** Habitus, lateral view **2** Terminalia, lateral view **3** Wing. Obs. The small crosssvein connecting stump vein with R_2+3_ is an anomaly **4** Terminalia, lateral view treated in hot 10% KOH **5** Sternite VIII **6** Gonocoxite and gonostylus **7** Apex of gonostylus **8** Aedeagus **9** Apex of aedeagus. Abbreviations: aed: aedeagus; cerc: cercus; ej apod: ejaculatory apodeme; epand: epandrium; goncx: gonocoxite; gonst: gonostylus; hypd: hypandrium; st VIII: sternite VIII.

**Figures 10–18.**
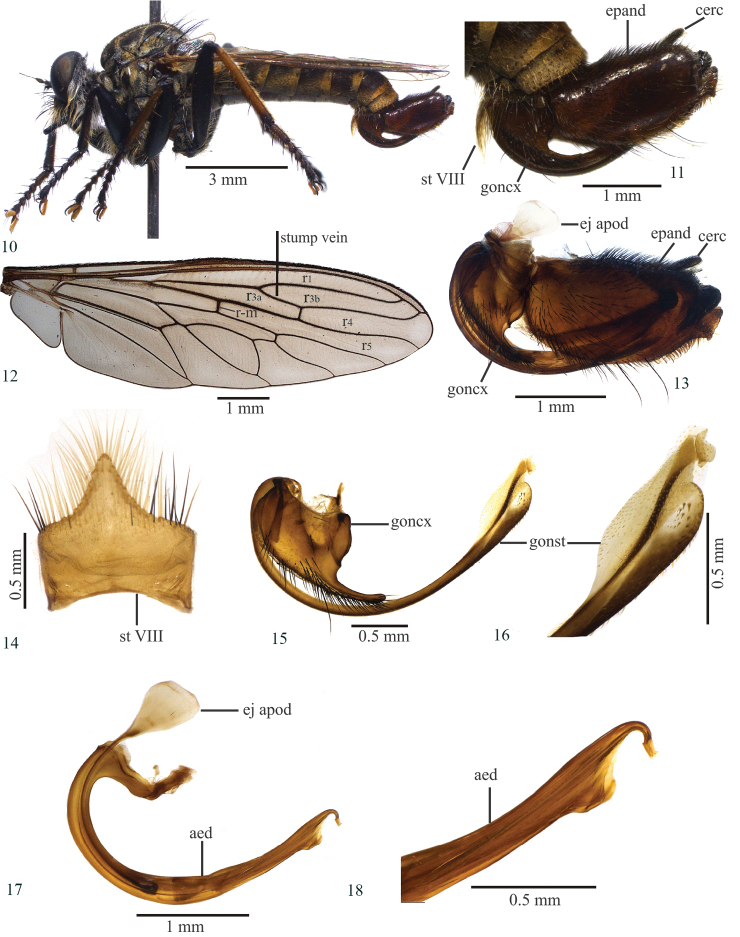
*Longivena digitata* sp. n. Holotype male. **10** Habitus, lateral view **11** Terminalia, lateral view **12** Wing **13** Terminalia, lateral view treated in hot 10% KOH **14** Sternite VIII **15** Gonocoxite and gonostylus **16** Apex of gonostylus **17** Aedeagus **18** Apex of aedeagus. Abbreviations: aed: aedeagus; cerc: cercus; ej apod: ejaculatory apodeme; epand: epandrium; goncx: gonocoxite; gonst: gonostylus; st VIII: sternite VIII.

**Figures 19–22.**
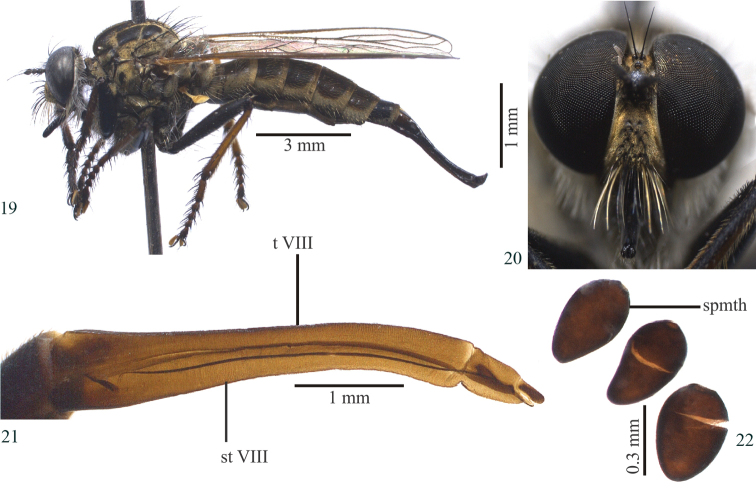
*Longivena digitata* sp. n. Paratype female: **19** Habitus, lateral view **20** Head, frontal view **21** Ovipositor, lateral view **22** Capsule of spermathecae; Abbreviations: spmth: capsule of spermathecae; t VIII: tergite VIII; st VIII: sternite VIII.

**Figures 23–31.**
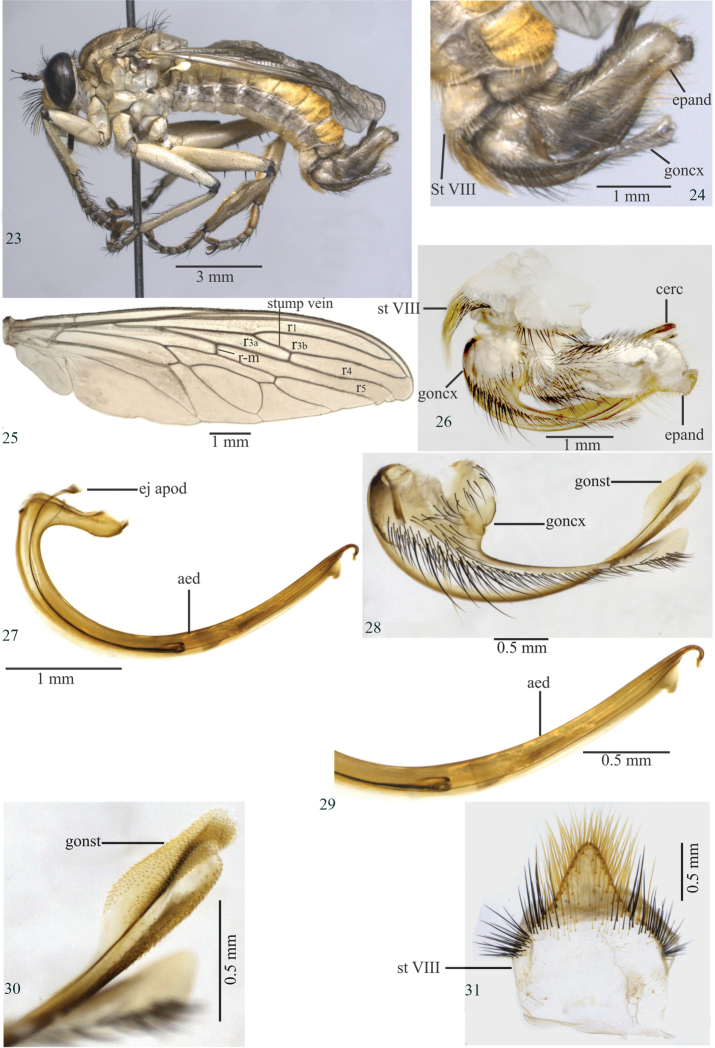
*Longivena flava* sp. n. Holotype male. **23** Habitus, lateral view **24** Terminalia, lateral view **25** Wing **26** Terminalia, lateral view treated in hot 10% KOH **27** Aedeagus **28** Gonocoxite and gonostylus **29** Apex of aedeagus **30** Apex of gonostylus **31** Sternite VIII. Abbreviations: aed: aedeagus; cerc: cercus; ej apod: ejaculatory apodeme; epand: epandrium; goncx: gonocoxite; gonst: gonostylus; st VIII: sternite VIII.

**Figures 32–40.**
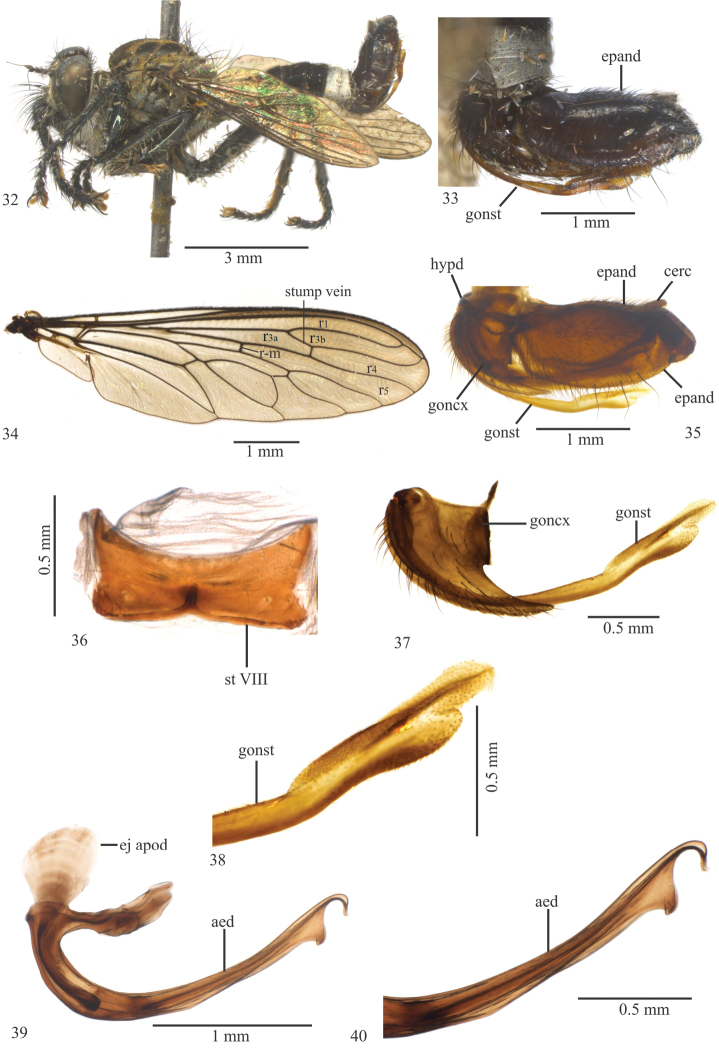
*Longivena limeiraoliverai* sp. n. Holotype male. **32** Habitus, lateral view **33** Terminalia, lateral view **34** Wing **35** Terminalia, lateral view treated in hot 10% KOH **36** Sternite VIII **37** Gonocoxite and gonostylus **38** Apex of gonostylus **39** Aedeagus **40** Apex of aedeagus. Abbreviations: aed: aedeagus; cerc: cercus; ej apod: ejaculatory apodeme; epand: epandrium; goncx: gonocoxite; gonst: gonostylus; hypd: hypandrium; st VIII: sternite VIII.

**Figures 41–49.**
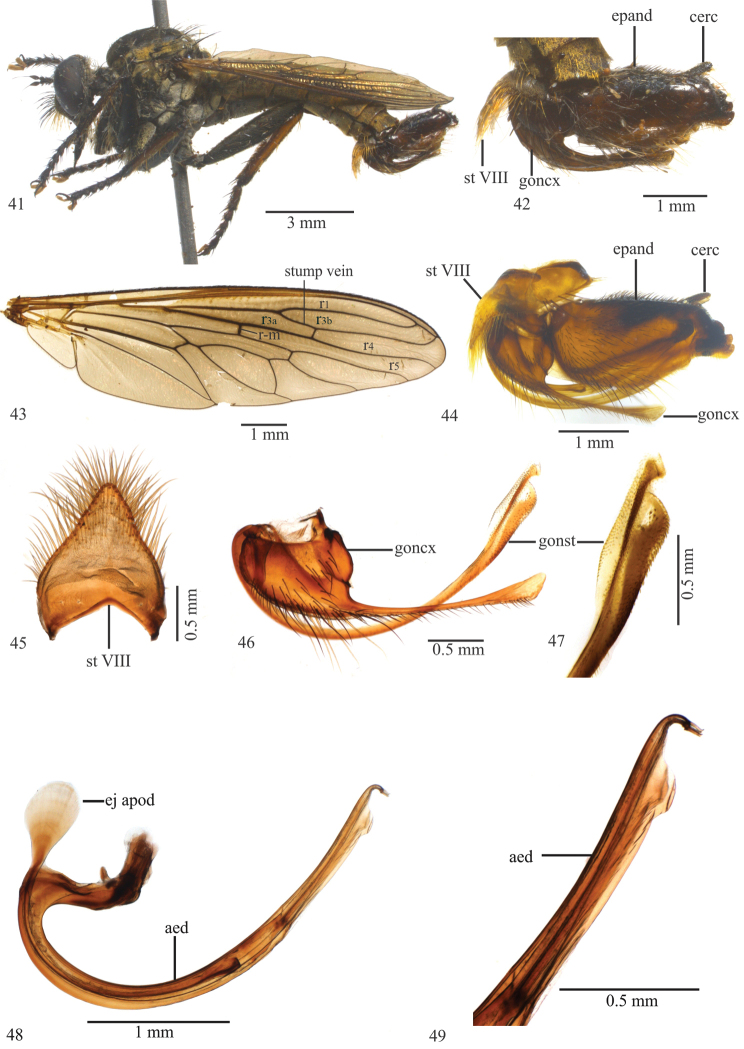
*Longivena spatulata* sp. n. Holotype male. **41** Habitus, lateral view **42** Terminalia, lateral view **43** Wing **44** Terminalia, lateral view treated in hot 10% KOH **45** Sternite VIII **46** Gonocoxite and gonostylus **47** Apex of gonostylus **48** Aedeagus **49** Apex of aedeagus. Abbreviations: aed: aedeagus; cerc: cercus; ej apod: ejaculatory apodeme; epand: epandrium; goncx: gonocoxite; gonst: gonostylus; st VIII: sternite VIII.

Head. Postpedicel oval, small, narrow distally; two to six setae, and two to four ocellar macrosetae; vertex black or yellowish, usually sparsely gold tomentose; face black or yellow, gold tomentose; proboscis black with thin and yellow ventral setae; labial setae yellowish; occipital setae white or yellowish. Thorax: two black notopleural macrosetae; one to two black supra-alar macrosetae; two black postalar macrosetae; no anatergal setae; thin, yellowish and black, discal scutellar setae yellowish or black, katatergal macrosetae yellowish; setae on posterior meron + metanepisternum yellowish. Wing: Without costal dilation; bifurcation of R_4+5_ beyond apex of discal cell; R_4_ supernumerary crossvein present, complete, reaching R_2+3_, as long as 1/5–1/6 of R_2+3_ length (Figs 3, 12, 21, 34, 43); microtrichia on posterior wing margin arranged in a single plane. Legs: Femora black or light yellow (Figs 1, 10, 19, 28, 32, 41); fore femur with yellow setae ventrally, occasionally with some black setae. Male abdomen: sternite VIII may be developed medioapically (Figs 9, 23, 45). Male terminalia: oblique to body axis (Figs 1, 10, 19, 32, 41); epandrium divided proximally and halves approximated medially; hypandrium dorsoventrally flatenned medially; hypandrium partially fused to base of epandrium; aedeagus directed dorsally (Figs 5, 17, 26, 39, 48). Female terminalia: Tergite VIII long and slender (Figs 28, 30). Tergite IX + X membranous medioapically; three oval and sclerotized spermathecal capsules (Fig. 31).

### Longivena bilobata sp. n.

Taxon classificationAnimaliaDipteraAsilidae

http://zoobank.org/D228DDAB-CB6A-4A0A-9459-4ED70BAC70B5

Figs 1–9

#### Diagnosis.

Femora wholly black (Fig. 1); hind tibia, anteriorly, with basal 4/5 yellow and apical 1/5 black with a brown stripe, and posteriorly with basal 2/3 yellow and apical 1/3 brown; basal margin of epandrium straight, apex bilobate in lateral view (Figs 2, 4); gonocoxite apex rounded (Fig. 6).

#### Male.

**Holotype.** Head. Antenna black; two setae and two long ocellar macrosetae; vertex, frons and face black, all sparsely golden tomentose; mystax with black and yellow setae; palpus with yellow basal setae and black apical setae; proboscis black with yellow ventral setae; labial setae yellowish; occiput black, silvery tomentose with white setae; ten black postocular macrosetae.

Thorax (Fig. 1). Antepronotum and postpronotum black, gold to brown tomentose; mesonotum black dorsally, golden laterally, with gold tomentose presutural and prescutellar spot; pleuron gray tomentose, except anepisternum gray and golden tomentose; scutellum gold tomentose. Chaetotaxy: Two black notopleural macrosetae; two black supra-alar macrosetae; two black postalar macrosetae; four pairs black presutural dorsocentral macrosetae; no anatergal setae; two black apical scutellar setae; discal scutellar setae black; katatergal macrosetae black; setae on posterior meron + metanepisternum yellowish.

Wing (Fig. 3). Brown. Crossvein r-m passes slightly beyond middle of discal cell; halter yellow.

Legs (Fig. 1). Femora wholly black. Fore tibia black; mid tibia dark brown to black anteriorly and yellow posteriorly; hind tibia, anteriorly, with basal 4/5 yellow and apical 1/5 black with a brown stripe, and with basal 2/3 yellow and apical 1/3 brown posteriorly. Fore femur with yellow ventral setae; mid femur with five black anteroventral setae, with one black apical seta dorsoposteriorly; hind femur with two yellow and one black macrosetae anteriorly, three yellow on basal 1/2 and three black macrosetae on apical 1/2 anteroventrally, and one apical black macrosetae posterodorsally; fore tibia with two black setae posteriorly; mid tibia with one long, black anteroventral seta; hind tibia with three black anterodorsal and two anteroventral setae; tarsomeres with black setae.

Abdomen. Tergites II–V dark brown, gold tomentose apically and laterally; tergites VI–VII golde tomentose; sternites I–V brown tomentose and sternites VI–VII golden tomentose. Sternite VIII not developed medioapically.

Terminalia (Figs 2, 4–9). Dark Brown to black (Fig. 2); epandrium basal margin straight, apex bilobate in lateral view (Figs 2, 4); gonostylus apex subtruncated (Figs 6, 7); gonocoxite apex rounded (Fig. 6); ejaculatory apodeme long and widened proximally in lateral view (Fig. 8); aedeagus with ventral projection directed dorsally and placed before folded apex (Figs 8, 9).

**Length.** Body 13.4 mm; wing 9.0 mm.

#### Female.

Unknown.

#### Variations

**(n= 5).** Body length 12.6–15 mm; wing length 8.3–10 mm; eight to nine black postocular macrosetae; two black, apical scutellar macrosetae; black and yellow katatergal macrosetae; one black supra-alar macrosetae; capitulum reddish; hind femur with two yellow setae on basal 1/2 and two black on apical 1/2 anteroventrally.

#### Etymology.

From Latin, *bi* = two + *lobus* = lobe, referring to the bilobate apex of the epandrium.

#### Holotype conditions.

Left detached wing mounted on microslides, terminalia placed in microvial with glycerin and pinned along with the specimen.

#### Distribution.

**Figure 50.**
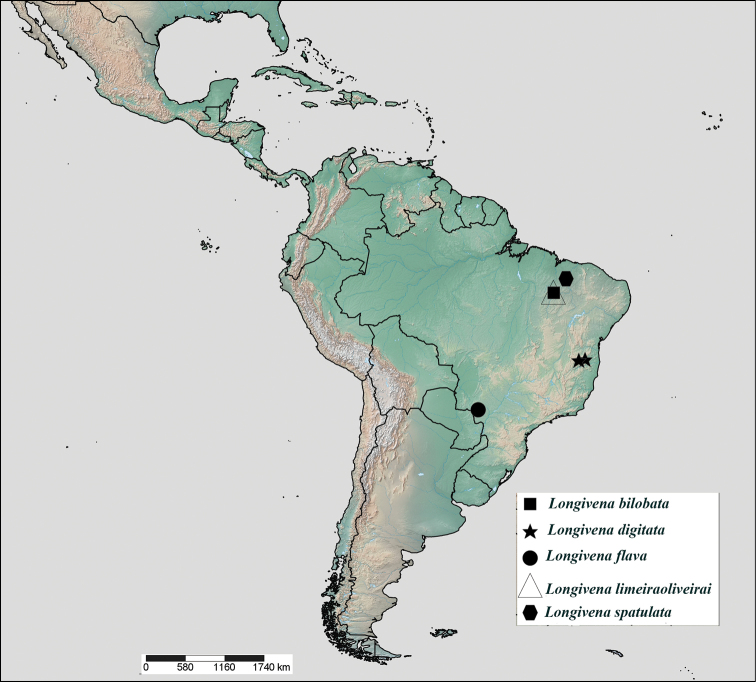
Distribution of *Longivena* gen. n. species.

**Brazil:** Maranhão (Fig. 50).

#### Type material examined.

**Holotype:** BRASIL, MA[ranhão], Mirador, Parque

Est.[adual] Mirador, Base do Mosquito [06°43'S 44°58'W] / Armadilha de Malaise, 04–08.ii.2011, F. Limeira–de–Oliveira / Holotype *Longivena bilobata* (♂ INPA).

**Paratypes:** Same data as holotype / Paratype *Longivena bilobata* (1♂ INPA; 1♂ CZMA); BRASIL, MA[ranhão], Mirador, Parque Est.[adual] Mirador, Base da Geraldina / Armadilha Luminosa, 08–13.iii.2008, F. Limeira–de–Oliveira, J.C. Silva / Paratype *Longivena bilobat* a (1♂ INPA); BRASIL, MA[ranhão], Mirador, Parque Est.[adual] Mirador, Posto avançado do Mel, 06°43'50"S, 44°58'59"W / Armadilha Luminosa 02–08.iv.2001, F. Limeira–de–Oliveira, G.A. Reis & M.S. Oliveira, cols. / Paratype *Longivena bilobata* (1♂ CZMA).

### Longivena digitata sp. n.

Taxon classificationAnimaliaDipteraAsilidae

http://zoobank.org/42183E59-2539-4278-8638-89EBCCBF7F34

Figs 10 –22

#### Diagnosis.

Six setae and two ocellar macrosetae; no apical scutellar seta; sternites VI–VIII brown, golden tomentose; tergites I–II with yellow setae laterally; sternite VIII developed medioapically, with black and yellow setae (Fig. 14); gonocoxite with a digitiform projection (Fig. 15); aedeagus with a trapezoidal process apically (Figs 17, 18). Female: abdominal tergites I–IV brown, golden tomentose laterally; tergite VII black, golden tomentose laterally; tergite VIII dark brown, long and slender (Figs 19, 21); three spermathecal capsules oval and sclerotized (Fig. 22).

#### Male.

**Holotype.** Head. Scape and pedicel black, postpedicel dark brown to black; six setae and two ocellar macrosetae; vertex black, sparsely golden tomentose; frons black; face black, golden tomentose; mystax mostly with black macrosetae and a few golden macrosetae on oral margin; palpus with yellow to brown setae; proboscis black with yellow ventral setae; labial setae yellowish; occiput black, sparsely gray tomentose, with yellow setae; eight to nine postocular macrosetae.

Thorax. Antepronotum golden, yellow to brown tomentose; postpronotum golden tomentose; mesonotum black, presutural and prescutellar spot golden and yellow tomentose; pleuron black, gray and golden tomentose. Chaetotaxy: two black notopleural macrosetae; one black supra-alar macrosetae; two black postalar macrosetae; two pairs of presutural and four pairs of postsutural dorsocentral macrosetae; no anatergal, neither apical scutellar setae; discal scutellar setae yellowish and blackish; katatergal macrosetae yellow; setae on posterior meron + metanepisternum yellowish.

Wing (Fig. 12). Brown. Crossvein r-m basal to middle of discal cell; halter yellow.

Legs (Fig. 10). Femora black; fore tibia black anteriorly and dorsally, brown ventrally, mixed black and brown posteriorly; mid tibia mostly brown, except for a black stripe anteriorly and black apex dorsally; hind tibia brown, black posteroapically; first tarsomere: fore black, mid dark brown and hind brown. Fore femur with only yellow ventral setae; mid femur with three black apical macrosetae, and yellow and black ventral setae; two yellow, basal and preapical, macrosetae posteriorly; hind femur with two yellow and one black macrosetae anteriorly, four yellow basal and two to three medioapical macrosetae anteroventrally; fore tibia with four yellow posteroventral setae on right leg and three yellow and one black apical setae on left leg; mid tibia with three yellow setae; hind tibia with five yellow setae dorsally, two to three black setae anteriorly, one yellow preapical seta ventrally; fore and mid tarsus with one yellow seta, hind tarsus with one to two yellow setae.

Abdomen. Tergites dark brown, golden tomentose; sternites VI–VIII brown, golden tomentose; sternite VIII developed medioapically, with black and yellow setae; tergites I–II with yellow setae laterally.

Terminalia (Figs 11, 13–18). Brown, except cercus and apex of epandrium black (Fig. 11). Basal margin of the epandrium rounded, apex truncated in lateral view (Figs 11, 13); gonostylus with spiniform setae subapically (Figs 15, 16); gonocoxite with digitiform projection and with black setae ventrally (Fig. 15); ejaculatory apodeme long and wide proximally in lateral view (Fig. 17); aedeagus with trapezoidal projection placed before the curved apex (Figs 17, 18).

**Length.** Body 12.2 mm; wing 8.1 mm.

#### Holotype conditions.

Two ocellar setae lost; some mystax setae broken; one left dorsocentral macroseta lost; cells r4 and r5 punctured apically; right mid tibia with one seta broken and another lost; right hind femur with two anteroventral setae broken and left hind femur with three anteroventral macrosetae broken. Left detached wing mounted on microslides, terminalia placed in microvial with glycerin and pinned along with the specimen.

**Variation (n= 12):** Body length 13.3–14.9 mm, wing 8.4–9.5 mm; three to seven ocellar setae; frons black, golden tomentose; five to seven postocular macrosetae; five pairs of postsutural dorsocentral macrosetae; one macrosetae and one, or two, apical scutellar setae; two black supra-alar macrosetae; halter stem yellow and capitulum reddish; mid femur with a black seta anteriorly; hind femur with three yellow macrosetae anteriorly, and four yellow and one to two anteroventrally; fore tibia with three to four yellow and one black setae posteroventrally; mid tibia with four yellow setae; hind tibia with two yellow setae anteriorly and one preapical ventrally; mid tarsus with black setae; tergites II–IV gray tomentose laterally; sternites II–VI gray tomentose.

**Female (Figs 19–22):** Similar to male, except for body length: 13.5–17.2 mm, wing 9.3–9.9 mm; six to eight postocular macrosetae; face dark brown, golden tomentose (Fig. 20); three to four pairs of postsutural dorsocentral macrosetae; one to two supra-alar macrosetae; no apical scutellar seta, except in one paratype that has one macroseta and one seta, and another that has only one seta; one paratype with fore basal tarsomere dark brown, mid and hind basal tarsomeres brown. Mid femur with one to three black macrosetae anteriorly (one paratype with three yellow macrosetae); hind femur with at most two yellow and one to two black macrosetae anteriorly (two paratypes with three yellow macrosetae, one of them with an additional black seta); hind femur with at most two black and four to five yellow macrosetae anteroventrally; fore tibia with three to four yellow setae posteroventrally (one paratype with two yellow and two black setae on left fore tibia); mid tibia with two to four yellow setae; hind tibia with four to five yellow setae dorsally (one paratype specimen with two yellow and one black setae anteriorly, one preapical and one yellow seta medioventrally); tarsomeres with only black setae, except for one paratype that has one yellow seta on fore and mid tarsomeres; hind tarsomere with one to two yellow setae; abdominal tergites I–VI brown, golden tomentose laterally; tergite VII black, golden tomentose laterally; tergite VIII dark brown, long and slender (Figs 19, 21); tergite IX + X membranous posteromedially; three spermathecal capsules oval and sclerotized (Fig. 22).

**Etymology:** From Latin *digitata* = digitate, finger-like, referring to the shape of the gonocoxite’s apex.

**Distribution. Brazil:** Bahia and Minas Gerais (Fig. 50).

**Type material examined. Holotype:** BRASIL, BA[hia], Encruzilhada, 15°32'25"S, 40°50'12"W, 10–12.xii.2007 / J.A. Rafael, P.C. Grossi & D.R. Parizotto col.[etores], Armadilha Luminosa, 800m / Holotype Longivena digitata (♂ INPA).

**Paratypes:** same data as holotype (4♀ INPA); BRASIL, MG [Minas Gerais], Berizal, Fazenda Veredão, 15°39'54"S, 41°39'56"W / 14.xii.2007, J.A. Rafael, P.C. Grossi & D.R. Parizotto col.[etores], Armadilha Luminosa, 850m / Paratype Longivena digitata (1♀ INPA); BRASIL, MG [Minas Gerais], Berizal, Fazenda Veredão, 15°39'54"S, – 41°39'56"W, 11.xii.2012, J.A. Rafael & E.J. Grossi, Arm. Luminosa, 850m / Paratype Longivena digitata (3♂, 1♀ INPA); BRASIL, BA[hia], Encruzilhada, 15°32'25"S, 40°50'12"W, 15.xii.2007 / J.A. Rafael & P.C. Grossi col.[etores], Armadilha Lumisona, 800m / Paratype Longivena digitata (2♂ INPA).

### Longivena flava sp. n.

Taxon classificationAnimaliaDipteraAsilidae

http://zoobank.org/333952F0-A70A-4443-9A16-8A4A5B43A5D5

Figs 23 –31

#### Diagnosis.

Antepronotum and postpronotum yellow; mesonotum yellowish (Fig. 23); pleuron yellow, except for black katepimeron (Fig. 23); legs whitish yellow, except for femora with black apex (Fig. 23); tergites yellow, with black dorsal maculae on tergites I–V (Figs 23, 24); sternites whitish to light yellow; male sternite VIII developed medioapically (Fig. 31); terminalia light yellow basally and brown to black on remaining area (Figs 23, 24).

#### Male.

**Holotype.** Head. Scape yellow with black apex, pedicel yellow, but black dorsally and apically, postpedicel black; two long ocellar macrosetae and seven long setae; vertex yellow, gold tomentose; frons and face yellow, gold tomentose; mystax with black and yellow macrosetae; palpus yellow with black and yellow setae; proboscis black, except for yellow labella with labial setae yellowish; occiput gold tomentose with yellow setae; eight to nine black postocular macrosetae.

Thorax (Fig. 23). Antepronotum and postpronotum yellow; mesonotum yellow; pleuron yellow, except for black katepimeron. Chaetotaxy: two black notopleural macrosetae; one to two black supra-alar macrosetae; two black postalar macrosetae; black dorsocentral macrosetae; no anatergal setae; no apical scutellar setae; discal scutellar setae yellowish, except for one black seta; katatergal macrosetae yellowish; setae on posterior meron + metanepisternum yellowish.

Wing (Fig. 3). Crossvein r-m basal to middle of discal cell; halter whitish-yellow.

Legs (Fig. 25). Light yellow, except apex femora black. Fore femur with thin and yellow ventral setae; mid femur with three black anterior setae; hind femur with three yellow anterior setae, two yellow and three black anteroventral setae, and preapical dorsal region with one black anterior macroseta and one yellow posterior macroseta; fore tibia with two to three yellow and one black posterior macrosetae; mid tibia with two long black anteroventral setae; hind tibia with three black and one yellow anterior setae; hind tibia with a group of short, yellow, spiniform setae, in a row from the base of the tibia and continuing until the fourth tarsomere; fore and mid tarsomeres with black setae; hind tarsomeres with mostly black setae, except for two yellow setae apically on the first tarsomere.

Abdomen (Fig. 23). Tergites yellow, with black dorsal maculae on tergites I–V; sternites whitish to light yellow; sternite VIII developed medioapically.

Terminalia (Figs 24, 26–31). Light yellow basally, remaining dark brown to black (Figs 23, 24). Epandrium with a truncate projection in lateral view (Figs 24, 26); gonocoxite with a long projection truncated apically (Fig. 28); gonostylus enlarged apically, with small spiniform setae (Fig. 30); ejaculatory apodeme long and narrow proximally in lateral view (Fig. 27); aedeagus with round ventral projection placed before the curved apex (Figs 27, 29).

**Length.** Body 12.2 mm; wing 9.2 mm.

#### Female.

Unknown.

#### Holotype conditions.

left wing slightly wrinkled; right katatergite and left posterior basalare sclerite punctured; right mid leg glued onto thorax; hind tibiae crushed medially. Right detached wing mounted on microslides, terminalia placed in microvial with glycerin and pinned along with the specimen.

#### Etymology.

From Latin *flava* = yellow, referring to the specimen’s color.

**Distribution. Brazil:** Mato Grosso do Sul (Fig. 50).

#### Type material examined.

**Holotype:** BRASIL, Mato Grosso do Sul, Dourados, 22°11'42.2"S, 54°58'35.6"W, 398m, 23.x–06.xi.2012 / Malaise, Teles Col., Amostra 15 / Holotype *Longivena flava* (♂ INPA).

### Longivena limeiraoliverai sp. n.

Taxon classificationAnimaliaDipteraAsilidae

http://zoobank.org/328692B6-E5FA-41BE-8F23-7746142FA8FB

Figs 32 –40

#### Diagnosis.

Two setae and four ocellar macrosetae; sternite VIII without projections (Fig. 36); epandrium basal margin rounded, apex subtriangular in lateral view, without projections (Figs 33, 35); gonostylus apex rounded with an apically rounded ventral projection (Figs 36, 37); gonocoxite tapering apically (Fig. 37); aedeagus apex with a subtriangular projection (Figs 39, 40).

#### Male.

**Holotype.** Head. Antenna black; two setae and four ocellar macrosetae; vertex, frons and face black, golden tomentose; mystax with black macrosetae; palpus with black setae; proboscis black with yellow, ventral setae; labial setae yellowish; occiput black, silver tomentose, sparse; white occipital setae; seven to nine black postocular macrosetae.

Thorax (Fig. 32). Antepronotum black, golden to brown tomentose; postpronotum black, golden tomentose; mesonotum black dorsally, golden laterally, presutural and prescutellar spot golden tomentose; pleuron gray tomentose, except for anepisternum black and sparsely gray tomentose; scutellum golden tomentose. Chaetotaxy: two black notopleural macrosetae; two black supra-alar macrosetae; three pairs black presutural dorsocentral macrosetae; no anatergal setae; three macrosetae and black, apical scutellar setae; black discal scutellar setae blackish; katatergal macrosetae yellow; setae on posterior meron + metanepisternum yellowish.

Wing (Fig. 34). Brown. Crossvein r-m passes slightly beyond middle of discal cell; halter yellow.

Legs (Fig. 32). Wholly black. Fore femur with yellow and black setae ventrally; mid femur with yellow setae ventrally, with one black apical macroseta posterodorsally; hind femur with four black setae and one black macroseta anteroventrally, and three black macrosetae anteriorly; fore tibia with four black setae posteriorly; mid tibia with three yellow setae posteriorly; hind tibia with two black macrosetae posteriorly; tarsomeres with black setae.

Abdomen. Tergites I–II black, gray tomentose on apical margin; tergites II–III with gray tomentose triangular macula; tergites IV–V wholly black and tergites VI–VII gray tomentose (Figs 32, 33); sternites I–V gray and brown tomentose and sternites VI–VII only gray tomentose; sternite VIII without projections (Fig. 36).

Terminalia. Brown to black (Figs 32, 33). Epandrium basal margin rounded, apex subtriangular in lateral view, without projections (Figs 33, 35); gonostylus apex rounded, with an apically rounded ventral projection (Figs 37, 38); gonocoxite tapering apically (Fig. 37); ejaculatory apodeme short and wide proximally in lateral view (Fig. 39); aedeagus with subtriangular process before curved apex (Figs 39, 40).

**Length.** Body 10.1 mm; wing 6.8 mm.

#### Holotype conditions.

Left detached wing mounted on microslides, terminalia placed in microvial with glycerin and pinned along with the specimen.

#### Female.

Unknown.

#### Variations

**(n= 3).** Body length 10.1–12.1 mm; wing 6.3–7.5 mm; four pairs black, presutural dorsocentral macrosetae; four macrosetae and two black apical scutellar setae; capitulum reddish brown; hind femur with two black macrosetae anteriorly; hind tibia with three black macrosetae posteriorly.

#### Etymology.

A patronym to the researcher Francisco Limeira–de–Oliveira, PhD at Universidade Estadual do Maranhão.

#### Distribution.

**Brazil:** Maranhão (Fig. 50).

#### Type material examined.

**Holotype:** BRASIL, MA[ranhão], Mirador, Parque

Est.[adual] Mirador, Povoado Pindaíba (Mel), 06°39'44"S, 45°01'37"W / Armadilha de Malaise, 01–05.vi.2011, F. Limeira–de–Oliveira, M.M. Abreu & J.S. Pinto Júnior / Holotype *Longivena limeiraoliverai* (♂ INPA).

**Paratypes:** same data as holotype (1♂ INPA); BRASIL, MA[ranhão], Mirador, Parque Est.[adual] Mirador, Base da Geraldina / Armadilha de Malaise, 20.v–02.vi.2007, F. Limeira–de–Oliveira, Cols / Paratype *Longivena limeiraoliverai* (1♂ CZMA); same data as holotype: 07–14.v.2010, J.C. Silva & M.M. Abreu / Paratype *Longivena limeiraoliverai* (1♂ CZMA).

### Longivena spatulata sp. n.

Taxon classificationAnimaliaDipteraAsilidae

http://zoobank.org/8293ED25-CC91-47BC-A821-98AC41243DCB

Figs 41 –49

#### Diagnosis.

four setae and two ocellar macrosetae; tergites V–VII brown; sternites I–IV black, gray tomentose; sternite VIII triangular, developed medioapically and with yellow setae (Fig. 45); epandrium basal margin rounded, apex with two truncated projections in lateral view (Figs 42, 44); gonocoxite with a long spatulated projection (Fig. 46).

#### Male.

**Holotype.** Head. Antenna black; four setae and two ocellar macrosetae; vertex black, sparsely golden tomentose; frons black; face black, golden tomentose; mystax with black setae on dorsal 1/2 of facial swelling and yellow setae on ventral 1/2; palpus with yellow setae on basal 1/2 and black setae on apical 1/2; proboscis black with yellow setae ventrally; yellow labial setae; occiput black, golden tomentose, sparse; yellow occipital setae; seven black and four yellow, thick, postocular macrosetae on each side of the head.

Thorax (Fig. 41). Antepronotum black, golden to brown tomentose; postpronotum black, golden tomentose; mesonotum black dorsally, golden laterally, presutural and prescutellar spot golden tomentose; pleuron gray tomentose, except for anepisternum black, sparsely golden tomentose; scutellum golden tomentose. Chaetotaxy: two black notopleural macrosetae; two black supra-alar macrosetae; two black postalar macrosetae; two pairs black presutural dorsocentral macrosetae; no anatergal setae; one pair of yellow apical scutellar macrosetae; katatergal macrosetae yellow; setae on posterior meron + metanepisternum yellowish.

Wing (Fig. 43). Brown. Crossvein r-m passes slightly beyond middle of discal cell; halter with yellow stem and dark red capitulum.

Legs (Fig. 41). Femora black; fore tibia black anteriorly, brown ventrally, and with basal 1/2 brown and apical 1/2 black posterodorsally; mid tibia with basal 2/3 brown and apical 1/3 black anteriorly, remaining brown (except apex black); hind tibia brown, black posteroapically; tarsomeres brown, except for black apical ones. Fore femur with only yellow setae ventrally; mid femur with a yellow macroseta anteromedially and two black macrosetae apically and one yellow apical seta posterodorsally; hind femur with three yellow macrosetae anteriorly, three yellow setae basally, and three black macrosetae apically, one black preapical macroseta anterodorsally and one yellow preapical macroseta posterodorsally; fore tibia with four yellow setae posteriorly; mid tibia with two black setae anteriorly and three yellow setae posteriorly; hind tibia with three yellow setae anteriorly and three posteriorly; tarsomeres with black setae, except for one yellow seta on the first tarsomere of the right mid leg.

Abdomen. Tergites I–IV black, gray tomentose, with brown apical margin, tergites V–VII brown; sternites I–IV black, gray tomentose; sternites V–VII gray and brown tomentose; sternite VIII triangular, medioapically developed and with yellow setae.

Terminalia (Figs 42, 44–49). Brown to black (Figs 41, 42). Epandrium basal margin rounded, apex with two truncated projections in lateral view (Figs 42, 44); gonostylus with apical indentation (Figs 46, 47); gonocoxite with long and spatulated projection, and medial margin with black and yellow setae (Fig. 46); ejaculatory apodeme long and wide proximally in lateral view (Fig. 48); aedeagus apex with trapezoidal projection (Figs 48, 49).

**Length.** Body 15.3 mm; wing 10.2 mm.

#### Holotype conditions.

Left hind leg lost; part of tergite I separated from thorax. Right detached wing mounted on microslides, terminalia placed in microvial with glycerin and pinned along with the specimen.

#### Female.

Unknown.

#### Etymology,

From Latin *spatulata* = spatulated, referring to the shape of the gonocoxite apex.

#### Distribution.

**Brazil:** Maranhão (Fig. 50).

#### Type material examined.

**Holotype:** BRASIL, MA[ranhão], Caxias [4°52'29"S, 43°20'49"W], Res.[erva] Ecol.[ógica] Inhamum, Lençol e Luz mista, 29–31.i.2006, F. Limeira–de–Oliveira / Holotype *Longivena spatulata* (♂ INPA).

### Identification key to males of *Longivena* gen. n.

**Table:** 

1	Sternite VIII developed medioapically (Figs 14, 31, 45)	**2**
–	Sternite VIII not developed medioapically (Figs 5, 36)	**4**
2	Gonocoxite without a long apical extension (Fig. 15)	***Longivena digitata* sp. n.** (Brazil: Bahia state and Minas Gerais)
–	Gonocoxite with a long apical extension (Figs 28, 46)	**3**
3	Body mainly light yellow (Fig. 23); sternite VIII with black and yellow setae, lateral and ventral margin somewhat straight (Fig. 31); epandrium apex with one truncated projection in lateral view (Figs 24, 26); ejaculatory apodeme narrow proximally in lateral view (Fig. 27); aedeagus with round ventral projection placed before the apex (Figs 27, 29); tip of aedeagal prongs oriented backwards, hook-like (Figs 27, 29)	***Longivena flava* sp. n.** (Brazil: Mato Grosso do Sul)
–	Body mainly brown to black (Fig. 41); sternite VIII with only yellow setae, lateral margin curved rounded and strongly concave anteriorly (Fig. 45); epandrium apex with two truncated projections in lateral view (Figs 42, 44); ejaculatory apodeme wide proximally in lateral view (Fig. 48); aedeagus with trapezoidal ventral projection placed before the apex (Figs 48, 49); tip of aedeagal prongs oriented ventrally (Figs 48, 49)	***Longivena spatulata* sp. n.** (Brazil: Maranhão)
4	Tergites II–III black with a triangular gray tomentose dorsal macula; tergites IV–V wholly black and tergites VI–VII wholly gray tomentose; epandrium basal margin rounded, apex subtriangular in lateral view, without projections (Figs 33, 35); ejaculatory apodeme widened proximally in lateral view (Fig. 39); gonocoxite with tapering apex (Fig. 37); gonostylus apex rounded, with an apically rounded ventral projection (Figs 37, 38)	***Longivena limeiraoliverai* sp. n.** (Brazil: Maranhão)
–	Tergites II–V dark brown, golden tomentose apically and laterally; tergites IV–VII dark brown wholly golden tomentose; epandrium basal margin straight, apex bilobate in lateral view (Figs 2, 4); ejaculatory apodeme long and wide proximally in lateral view (Fig. 8); gonocoxite apex rounded (Fig. 6); gonostylus apex subtruncated (Figs 6, 7)	***Longivena bilobata* sp. n.** (Brazil: Maranhão)

## Discussion

*Longivena* gen. n. is morphologically similar to the genera of the artificial *Efferia* group and according to the key published by Artigas and Papavero (1997b), the genus keys with *Diplosynapsis* Enderlein, 1914 (step 4) because both genera possess a closed and petiolate cell r3. The species included in *Longivena* gen. n. are similar to the species of *Diplosynapsis*, but *Diplosynapsis* has a short stump vein (supernumerary crossvein) on R_4_, not reaching the base of vein R_2+3_, and some specimens bear anatergal setae whereas *Longivena* gen. n. has a long stump vein on R_4_ reaching the base of R_2+3_, as long as 1/5–1/6 of R_2+3_ length (Figs 3, 12, 21, 34, 43) and no anatergal setae.

The senior author included only *Longivena* gen. n., but not *Diplosynapsis* (no specimens of both sexes were available for study), in the cladistic analysis of Asilinae (Vieira *et.al*. in prep.). *Longivena* gen. n. forms a clade together with *Albibarbefferia* Artigas & Papavero, 1997, *Aristofolia* Ayala-Landa, 1978, *Carinefferia* Artigas & Papavero, 1997, *Ctenodontina* Enderlein, 1914, *Efferia* Coquillett, 1893, *Eichoichemus* Bigot, 1857, *Eicherax* Bigot, 1857, *Eraxasilus* Carrera, 1954, *Lecania* Macquart, 1838, *Nerax* Hull, 1962, *Pogonioefferia* Artigas & Papavero, 1997, *Porasilus* Curran, 1934 and *Triorla* Parks, 1968: ((*Ctenodontina* (*Lecania* (*Lecania* + *Eraxasilus*))) (*Eicherax* (*Triorla* (*Albibarbefferia* (*Porasilus* (*Carinefferia* (*Longivena* + *Eichoichemus*) (*Nerax* (*Aristofolia* + *Pogonioefferia*) *Efferia*))))))).

*Longivena* gen. n. and *Eichoichemus* are sister taxa sharing the synapomorphy: suture between labella and prementum strongly appressed, situated dorsally and five additional apomorphic character states: anterior tentorial pits small, slitlike, inconspicuous, ventrally located; presutural acrostichal setae in regular rows; postmetacoxal bridge absent, postmetacoxal area entirely membranous; stump vein supernumerary crossvein on R_4_ present, length intermediate (1/5–1/6 of vein R_2+3_); costal section on between tip of R_5_ and tip of M_1_ shorter than costal section between tips of R_1_ and R_5_.

**Biology.** Little is known about the biology of species of *Longivena* gen. n.. The species were collected with either light or Malaise traps. Only the female of *Longivena digitata* has been collected to this day. It is expected that with future collecting efforts, new species of *Longivena* gen. n. will be found as well as the females of the species described in this paper.

**Distribution.** The species of *Longivena* gen. n. were found in the Caatinga (*Longivena digitata* sp. n.) and Cerrado (*Longivena bilobata* sp. n., *Longivena flava* sp. n., *Longivena limeiraoliverai* sp. n. and *Longivena spatulata* sp. n.) habitats or environments. Caatinga is a region characterized by arboreal or bushy forests, having mainly small trees and bushes which bear spines and some xerophytic characteristics, average annual precipitation below 800 mm and, at most, a 0.5 aridity index (Prado 2003) and Cerrado biomes is a region characterized by a savannah-like vegetation, with a seasonal climate, with heavy rains between the months of October and March and a long dry period between June and September (Harley et al. 2005).

## Supplementary Material

XML Treatment for Longivena

XML Treatment for Longivena bilobata

XML Treatment for Longivena digitata

XML Treatment for Longivena flava

XML Treatment for Longivena limeiraoliverai

XML Treatment for Longivena spatulata
